# Enzymatic Synthesis of Glucose-Based Fatty Acid Esters in Bisolvent Systems Containing Ionic Liquids or Deep Eutectic Solvents

**DOI:** 10.3390/molecules21101294

**Published:** 2016-09-27

**Authors:** Kai-Hua Zhao, Yu-Zheng Cai, Xiao-Sheng Lin, Jun Xiong, Peter J. Halling, Zhen Yang

**Affiliations:** 1 College of Life Sciences and Oceanography, Shenzhen Key Laboratory of Microbial Genetic Engineering, Shenzhen University, Shenzhen 518060, Guangdong, China; zkh09023138@163.com (K.-H.Z.); LinXS3555@163.com (X.-S.L.); 2 College of Life Sciences and oceanography, Shenzhen Key Laboratory of Marine Bioresources and Ecology, Shenzhen University, Shenzhen 518060, Guangdong, China; cai_yz@cff-boton.com (Y.-Z.C.); x-xiongjun@foxmail.com (J.X.); 3 WestCHEM, Department of Pure & Applied Chemistry, University of Strathclyde, Glasgow G1 1XL, UK; p.j.halling@strath.ac.uk

**Keywords:** sugar fatty acid esters, ionic liquids, deep eutectic solvents, lipase, alkyl glucoside, esterification, transesterification

## Abstract

Sugar fatty acid esters (SFAEs) are biocompatible nonionic surfactants with broad applications in food, cosmetic, and pharmaceutical industries. They can be synthesized enzymatically with many advantages over their chemical synthesis. In this study, SFAE synthesis was investigated by using two reactions: (1) transesterification of glucose with fatty acid vinyl esters and (2) esterification of methyl glucoside with fatty acids, catalyzed by Lipozyme TLIM and Novozym 435 respectively. Fourteen ionic liquids (ILs) and 14 deep eutectic solvents (DESs) were screened as solvents, and the bisolvent system composed of 1-hexyl-3-methylimidazolium trifluoromethylsulfonate ([HMIm][TfO]) and 2-methyl-2-butanol (2M2B) was the best for both reactions, yielding optimal productivities (769.6 and 397.5 µmol/h/g, respectively) which are superior to those reported in the literature. Impacts of different reaction conditions were studied for both reactions. Response surface methodology (RSM) was employed to optimize the transesterification reaction. Results also demonstrated that as co-substrate, methyl glucoside yielded higher conversions than glucose, and that conversions increased with an increase in the chain length of the fatty acid moieties. DESs were poor solvents for the above reactions presumably due to their high viscosity and high polarity.

## 1. Introduction

The development of new eco-friendly surfactants has been an ongoing issue. Sugar fatty acid esters (SFAEs) are produced from renewable resources such as sugars and fatty acids. They are biodegradable, odorless, non-irritating, and non-toxic, and broad applications in the food, cosmetic, and pharmaceutical industries have been found [1,2,3,4].

Since the first paper proposing the lipase-catalyzed acylation of sugars with activated carboxylic esters in organic solvents [5], this one-step enzymatic strategy has been extended to SFAE synthesis, offering a promising means of cleaner production and hence acquiring significant popularity [6,7]. When catalyzed by a lipase (EC 3.1.1.3), sugars can be acylated to produce an SFAE by esterification with fatty acids or transesterification with active carboxylic esters. Both reactions are required to perform in nonaqueous media to prevent any possible hydrolysis of the ester product. The enzymatic approach has been proven to be superior to the currently dominating chemical synthesis in terms of mild reaction conditions, simple operational procedures, excellent regioselectivity, high productivity, and easy product separation. Enzymatic synthesis of SFAEs have been carried out in organic solvents [6] and in solid phase with addition of organic solvents as adjuvants [8,9].

Despite the above obvious advantages, finding a solvent able to solubilize both the sugar and the acyl donor, while also being compatible with the enzyme, remains a serious problem. Unprotected sugars are soluble only in a few hydrophilic organic solvents such as pyridine and dimethylformamide, which are, however, poor solvents for lipase activity [10]. Two strategies have been pursued to solve this problem: one strategy utilizes chemical modification on the substrate, typically the sugar, to make it more hydrophobic and hence more soluble [11,12,13,14,15]; the other strategy is based on solvent engineering. In this second strategy, ionic liquids (ILs) have shown great potential as a green alternative reaction medium to conventional organic solvents for sugar ester synthesis, promoting a significant enhancement in sugar solubility, enzymatic reactivity, and regioselectivity [7]. Here, ionic liquids are organic salts that are liquid at ambient temperature. Several research groups have carried out enzymatic SFAE synthesis in IL systems [16,17,18,19,20,21,22]. Meanwhile, special attention should be given to deep eutectic solvents (DESs), a new type of IL-related potentially green solvent prepared by complexation of a quaternary ammonium salt (e.g., choline chloride) with a hydrogen-bond donor (e.g., amide, amine, alcohol, and carboxylic acid) [23]. DESs have been found to possess some attractive IL-like solvent properties, such as low melting point, low volatility, high thermal stability, high solubility for various substances, and the “designer solvent” property. They have also shown some advantages over ILs, such as a much lower price, easier preparation with high purity, and superior environmental friendliness [24]. Although DESs have shown potential as either a solvent or co-solvent for a few biocatalytic applications (for review see [24]), only one application has so far been reported in terms of enzymatic sugar ester synthesis that was carried out in DES systems [25].

A recent study of ours [26] has demonstrated that, for the Novozym 435-catalyzed transesterification of glucose with vinyl laurate, 1-hexyl-3-methylimidazolium trifluoromethylsulfonate ([HMIm][TfO]) yielded the highest conversion among all 16 ILs tested, and the use of a bisolvent system composed of this IL and 2-methyl-2-butanol (2M2B) was favorable, as compared to the use of either the pure IL or pure 2M2B. Here, both the IL and 2M2B were completely miscible with each other. In the present work, enzymatic SFAE synthesis was further investigated by carrying out both esterification and transesterification reactions that are catalyzed by the two commonly used commercial lipases from Nozozymes, Novozym 435, and Lipozyme TLIM. The major goals of this study were (1) to further investigate the solvent effect on SFAE synthesis by using IL/2M2B or DES/2M2B bisolvent systems for the two synthetic reactions; (2) to examine the substrate effect by comparing the use of methyl glucoside vs. glucose as the acyl acceptor and the use of fatty acids vs. their vinyl esters with different chain lengths as the acyl donor; and (3) to optimize the conversion by single-factor experiments and response surface methodology (RSM).

## 2. Results and Discussion

### 2.1. IL and DES Screening

**Scheme 1:**
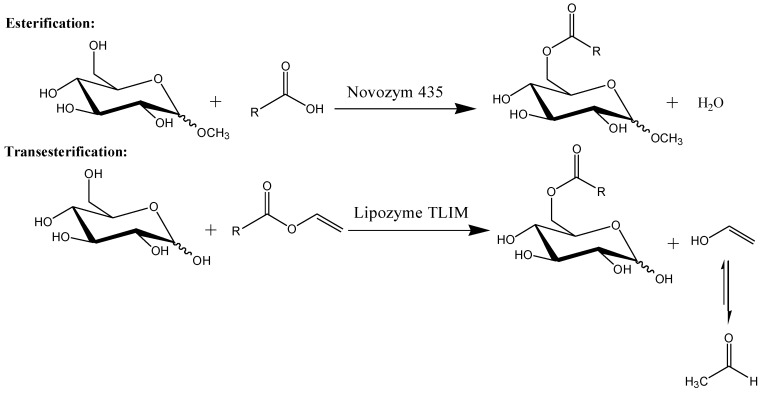
The two reactions used for sugar fatty acid ester (SFAE) synthesis in this study: (1) esterification of methyl glucoside with fatty acids, catalyzed by Novozym 435; (2) transesterification of glucose with fatty acid vinyl esters, catalyzed by Lipozyme TLIM.

Both Lipozyme TLIM and Novozym 435 are able to catalyze the synthesis of glucose laurate, either through transesterification of glucose and vinyl laurate or through esterification of methyl glucoside and lauric acid. The product was identified as a mono-ester [27]. As no systematic research has been carried out regarding the use of IL or DES as solvents for these two specific reactions catalyzed by the two enzymes, respectively (Reactions 1 and 2 in Scheme 1), 14 different ILs were screened for the two reactions, and 14 DESs for the second reaction.

The conversions obtained by the two synthetic reactions in 14 different ILs are reported in Table 1. For both reactions, ILs carrying hydrophilic anions (e.g., TfO^−^ and BF_4_^−^) are superior to those holding hydrophobic ones (e.g., PF_6_^−^ and Tf_2_N^−^) in promoting the synthesis, with [BMIm][TfO] and [HMIm][TfO] contributing the highest conversions, while the three MeSO_4_^−^-containing ILs contributing the lowest. This is in good agreement with our previous study [26], suggesting that both lipases have a similar preferences regarding the solvent selection. An in-depth discussion regarding the correlation between the solvent properties of ILs and the conversions of the enzymatic SFAE synthesis has been given in [26]. In our subsequent experiments, [HMIm][TfO] was selected, and a bisolvent system composed of this IL and 2M2B was employed as the reaction medium for the two reactions displayed in Scheme 1. Our recent study demonstrated that, for Novozym 435-catalyzed acylation of glucose with vinyl laurate, a higher conversion is achieved in this bisolvent system than in pure IL or pure 2M2B [26].

**Figure 1:**
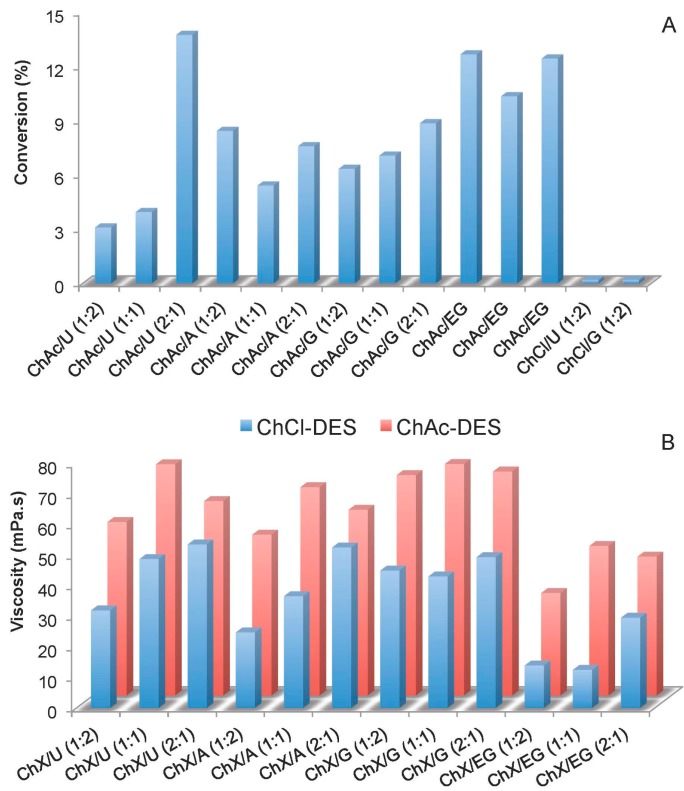
Conversions obtained in the DES/2M2B (1/9, *v/v*) bisolvent system (**A**) and viscosities of DESs containing 10% water (**B**). For reaction conditions used in A, a reaction mixture containing 0.054 g of glucose (corresponding to 0.3 mole/L), 0.3 M of vinyl laurate, 100 mg of molecular sieves, and 20 mg of Lipozyme TLIM in 1-mL DES/2M2B (0.1/0.9, *v/v*) was placed in an incubator/shaker with agitation of 300 rpm at 60 °C for 24 h. The 10% water contents in all the DESs used in (**B**) were determined by Karl Fischer titration with a Metrohm 831 KF coulometer. Here ChX refer to ChCl or ChAc, U, A, G and EG refer to urea, acetamide, glycerol and ethylene glycol, respectively.

As for DES screening, because of their high viscosities, 14 different DESs were each mixed with 2M2B to form a bisolvent system (DES/2M2B, 0.1:0.9, *v*/*v*) for the Lipozyme TLIM-catalyzed transesterification reaction (Reaction 2 in Scheme 1). One can see from Figure 1A that results obtained in all these DES systems were not encouraging. Comparatively, choline acetate (ChAc)-based DESs yielded higher conversions, while a negligible amount of the glucose laurate product was produced in the two choline chloride (ChCl)-based DES/2M2B solutions. Among the 14 DESs tested, ChAc/urea (U) (2:1) and the 3 ChAc/ethylene glycol (EG) DESs are the four that yielded the highest conversions.

So far, only one report has been given about the use of a DES as a solvent for enzymatic sugar ester synthesis [25]. The authors attempted to carry out Novozym 435-catalyzed transesterification of glucose and vinyl hexanoate in six ChCl-based DESs, but only two (i.e., ChCl/U and ChCl/glucose were successful and a low yield of glucose hexanoate was produced. Our study can serve as another proof of this concept, offering a new type of DES (i.e., ChAc-based) and a new enzyme (i.e., Lipozyme TLIM) for sugar ester synthesis. In support of this, our previous study has manifested that ChAc-based DESs were superior to the ChCl-based ones in activating and stabilizing *Penicillium expansum* lipase [28].

It is suspected that the poor production in the DES system may be related to the high viscosity of the solvent. Figure 1B presents the viscosity data for all 24 DESs prepared in this study: 12 ChCl-based and 12 ChAc-based. Plotting the conversions obtained in Figure 1A against the viscosities of those 12 ChAc-based DESs used did show a very rough correlation (plot not shown). However, the fact that the two ChCl-based DESs yielded rather poor conversions cannot simply be blamed for their viscosities, because ChAc-based DESs are generally more viscous (Figure 1B). Other solvent properties (such as high polarity, surface tension, water activity, etc.) may also be responsible for the poor yields. For instance, the low water content present in the reaction system might be part of the reasons to account for this. Durand et al. [29] have observed that an immobilized lipase was inactive in a pure ChCl/U DES, but an almost complete conversion was obtained when a 10% *v/v* of water was added to the reaction system.

### 2.2. Methyl Glucoside vs. Glucose

In this study, methyl glucoside was used in place of glucose as the starting material, in the hope of improving the substrate solubility in the solvent and in turn the conversion. As shown in Table 2, the solubility of methyl glucoside is indeed higher than that of glucose in both ILs ([HMIm][TfO]) and 2M2B and their mixtures. One can also see from Table 2 that, as the IL proportion increased in the IL/2M2B mixtures, both glucose and methyl glucoside became more soluble, again illustrating the benefit of using ILs as solvents for sugar ester synthesis. In fact, one of the advantages of using ILs as reaction media lies in their ability to dissolve a broad range of materials including sugars [30].

**Figure 2:**
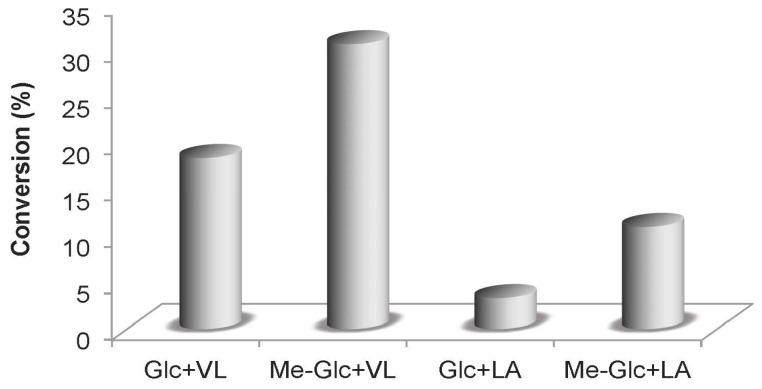
Comparison of the conversions obtained by transesterification and esterification with glucose and methyl glucoside as the co-substrates. Reaction conditions: 0.3 M of glucose (Glc) or methyl glucoside (Me-Glc), 0.3 M of vinyl laurate (VL) or lauric acid (LA), 100 mg of molecular sieve, 33.9 mg of Lipozyme TLIM, and 1 mL of 2M2B, 55 °C, 21 h.

Further, the parallel experiment shown in Figure 2 demonstrates that, for both transesterification and esterification reactions, the use of methyl glucoside as the co-substrate always yielded a higher conversion relative to the use of glucose. By studying the Novozym 435-catalyzed transesterification with methyl hexanoate in 2M2B to synthesize amino SFAEs, Pöhnlein et al. [31] have reported that reactions with a more hydrophobic sugar (*N*-butyryl-glucosamine, GlcNBu) exhibited significantly higher reaction rates and yields than those with *N*-acetyl-glucosamine (GlcNAc), a more hydrophilic sugar. Adelhorst et al. [11] have also noticed that, for esterification with fatty acids in a solvent-free system, ethyl glucoside reacted more slowly than propyl and butyl glucoside, but considerably faster than methyl glucoside or glucose. All of these experiments strongly suggest that SFAE synthesis is facilitated by utilizing a glycoside carrying an alkyl group as aglycon, which may be related to an improved substrate solubility, a better compatibility of the substrate with the enzyme’s active site, or both.

One can also see from Figure 2 that, for both glucose and methyl glucoside to be used, a higher conversion was obtained by transesterification with vinyl laurate than by esterification with lauric acid. The major reason for this is simply because of the formation of the unstable enol product, its tautomerization driving the reaction forward.

For subsequent experiments, esterification of methyl glucoside with palmitic acid, catalyzed by Novozym 435, and transesterification of glucose with vinyl laurate, catalyzed by Lipozyme TLIM, were both investigated in the [HMIm][TfO]/2M2B bisolvent system, and the effect of the fatty acid chain length was examined for both reactions.

### 2.3. Esterification of Methyl Glucoside and Palmitic Acid, Catalyzed by Novozym 435

**Figure 3:**
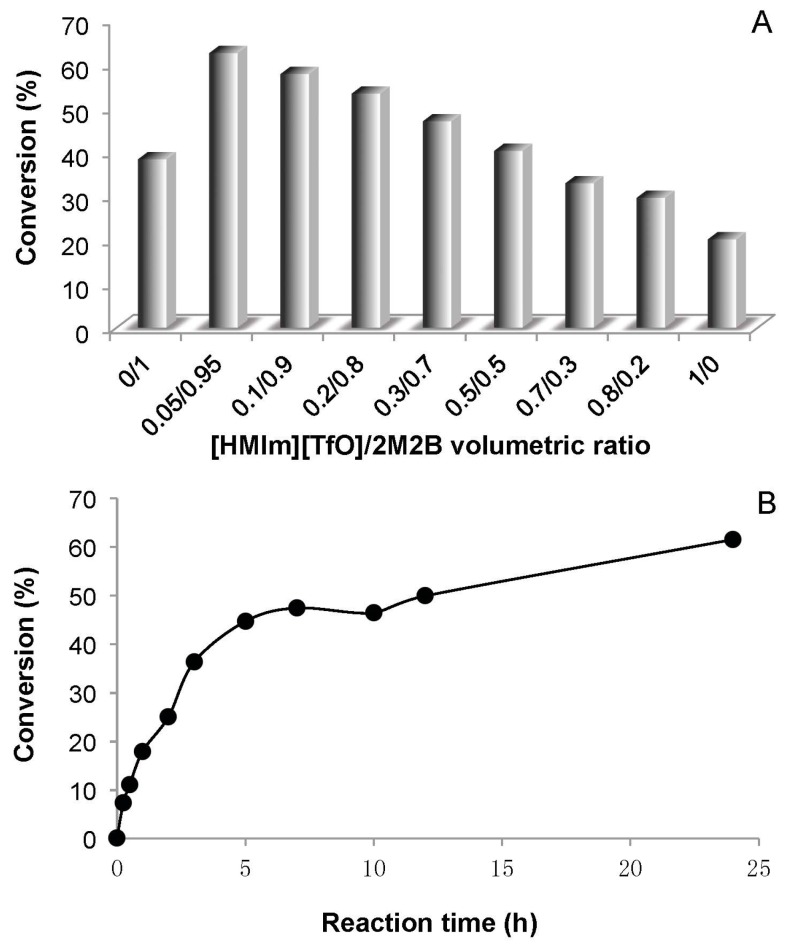
Novozym 435-catalyzed esterification of methyl glucoside and palmitic acid in the [HMIm][TfO]/2M2B bisolvent system. (**A**) Effect of IL/2M2B volumetric ratio on the conversions obtained in 21 h; (**B**) Time course of the reaction under optimal conditions: 0.3 M of methyl glucoside and palmitic acid, 150 mg of molecular sieve, 10 mg of Novozym 435, and 1.0 mL of solvent ([HMIm][TfO]/2M2B, 0.05:0.95, *v/v*) at 45 °C.

The esterification reaction was first performed in 2M2B, and the optimal reaction temperature and enzyme dosage were determined to be 45 °C and 10 mg, respectively. When the reaction was conducted in the [HMIm][TfO]/2M2B bisolvent system, the optimal volumetric ratio for the two co-solvents was 0.05:0.95 (Figure 3A), implying that the esterification reaction also prefers the use of the IL/2M2B bisolvent system to the use of either pure IL or pure 2M2B as the reaction medium. Under these optimal conditions, a conversion of 61.6% was achieved within 24 h (Figure 3B), which is translated to a superior specific productivity of 796.6 µmol/h/g. So far no data have been reported regarding the use of IL systems for lipase-mediated esterification reactions between alkyl glucoside and fatty acid to synthesize SFAEs.

### 2.4. Transesterification of Glucose and Vinyl Laurate, Catalyzed by Lipozyme TLIM

**Figure 4:**
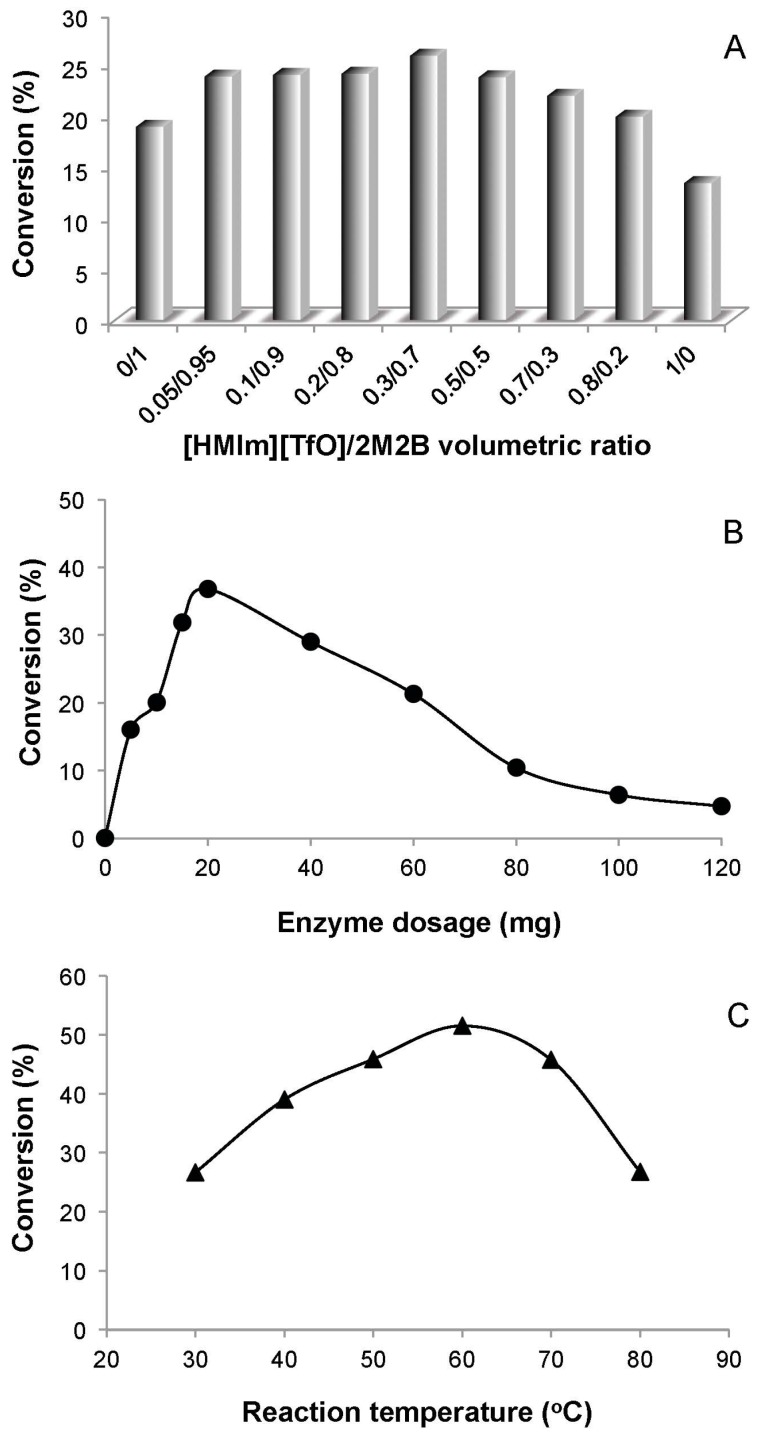
Lipozyme TLIM-catalyzed transesterification of glucose and vinyl laurate in the [HMIm][TfO]/2M2B bisolvent system: Effect of IL/2M2B volumetric ratio (**A**); enzyme dosage (**B**); and reaction temperature (**C**) on the conversions obtained in 24 h.

Here, Lipozyme TLIM was used as the catalyst while the reaction was also carried out in the [HMIm][TfO]/2M2B bisolvent system. The impacts of the affecting factors (i.e., IL/2M2B volumetric ratio, enzyme dosage, and reaction temperature) had been examined in order to work out the optimum for each condition. The optimal values turned out to be 3:7 (*v/v*), 20 mg and 60 °C, respectively (Figure 4). Under these conditions, the conversion obtained at 24 h was significantly enhanced from 26.8% (Table 1) to 46.4%. This experiment confirms the superiority of using the IL/2M2B bisolvent system instead of using the IL or 2M2B alone as the effect was observed not only for Novozym 435 but also for Lipozyme TLIM.

Based on the above single-factor results, response surface methodology (RSM) with a four-factor-three-level Box-Behnken design (BBD) was employed for modeling and optimization of the enzymatic synthesis of glucose laurate. The four factors (i.e., enzyme dosage, vinyl laurate /glucose (VL/Glc) molar ratio, reaction time, and 2M2B/IL volumetric ratio) and their varying levels are listed in Table 3. A total of 30 runs were carried out, among which six were at the central point. The model has been demonstrated to be valid, well reflecting the influence of each variable and their interactions on the conversion in the following polynomial Equation (1):

*Y = −161.08 − 1.22A − 12.28B + 16.58C + 11.78D + 2.22AB + 0.09AC + 0.14AD + 0.56BC + 0.06BD + 0.21CD − 0.04A^2^ − 15.12B^2^ − 0.41C^2^ − 3.10D^2^* (1)

where *Y* is the predicted conversion (%), while *A*, *B*, *C*, and *D* refer to enzyme dosage (mg), VL/Glc molar ratio, reaction time (h), and 2M2B/IL volumetric ratio, respectively.

**Figure 5:**
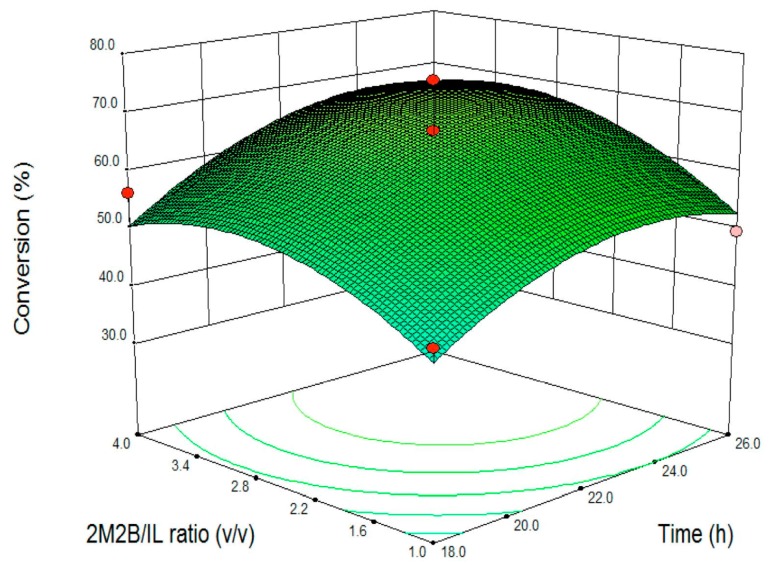
Response surface plot of conversions as a function of the 2M2B/IL volumetric ratio and reaction time (h).

One of the 3D response surfaces with contour plots is depicted in Figure 5. A maximal conversion of 99.2% was predicted by the model with a set of reaction conditions suggested: 33.9 mg (enzyme dosage), 1.4:1 (VL/Glc molar ratio), 20.9 h (reaction time), and 3.6:1 (2M2B/IL volumetric ratio). Three tests were done under these conditions, and an average conversion of 94.0% ± 1.1% was obtained, which is reasonably close to the predicted value. The specific productivity was 397.5 µmol/h/g, much higher than those obtained through enzymatic transesterification in IL systems after optimization by RSM [16,18,20,26] and those obtained in organic solvents [12,31,32,33,34].

### 2.5. Effect of Chain Length of the Acyl Donor 

**Figure 6:**
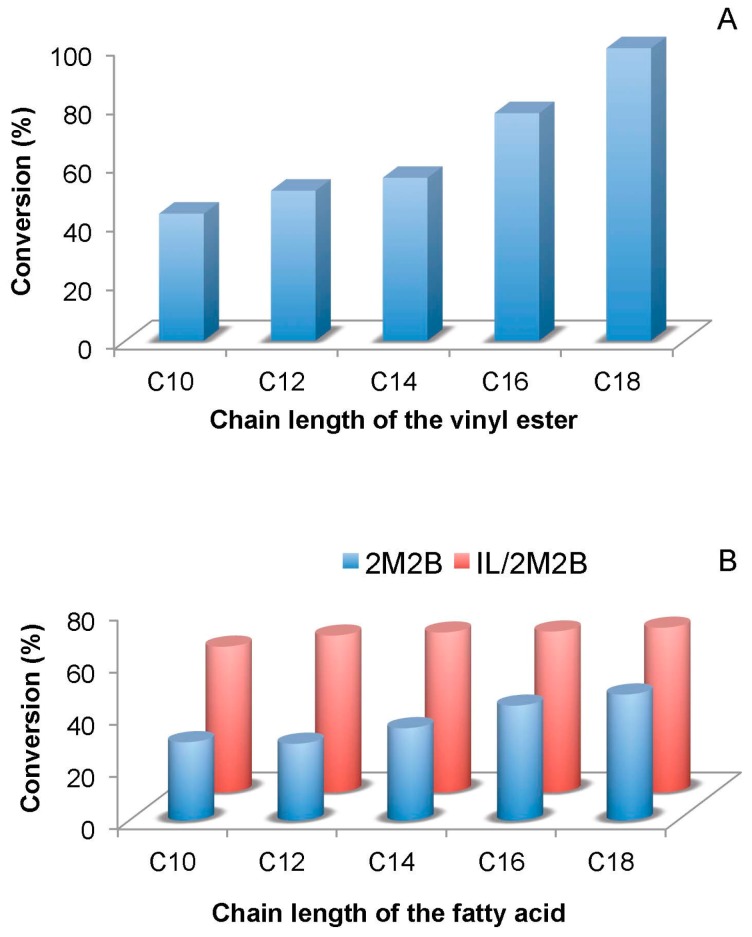
Variation of conversion with fatty acid chain length: (**A**) Lipozyme TLIM-catalyzed transesterification of glucose with various fatty acid vinyl esters for 7 h in [HMIm][TfO]/2M2B bisolvent system (3:7, *v/v*); (**B**) Novozym 435-catalyzed esterification of methyl glucoside with fatty acids with different chain lengths in 2M2B for 21 h and in [HMIm][TfO]/2M2B bisolvent system (0.05:0.95, *v/v*) for 24 h. C10, C12, C14, C16, and C18 refer to vinyl esters or acids of caprate, laurate, myristate, palmitate, and stearate, respectively. Reaction conditions for (A): 0.3 M of glucose, 0.3 M of vinyl ester, 100 mg of molecular sieves, 33.9 mg of Lipozyme TLIM, and 1.0 mL of IL/2M2B (0.2:0.8, *v/v*), 55 °C, 7 h. Conditions used for (**B**) in 2M2B: 0.3 M of methyl glucoside, 0.3 M of fatty acid, 100 mg of molecular sieve, 100 mg of Novozym 435, and 1.0 mL of 2M2B, 45 °C, 21 h. Conditions used for (**B**) in IL/2M2B bisolvent system: 0.3 M of methyl glucoside, 0.3 M of fatty acid, 150 mg of molecular sieve, 10 mg of Novozym 435, and 1.0 mL of [HMIm][TfO]/2M2B (0.05:0.95, *v/v*), 45 °C, 24 h.

Because the chain length of the acyl donor has a significant impact on the hydrophilic/hydrophobic balance of the SFAE to be produced, it is necessary to examine the SFAE synthesis using fatty acids or their vinyl esters with varying chain lengths. When glucose was acylated with a series of fatty acid vinyl esters through Lipozyme TLIM-mediated transesterification in the IL/2M2B bisolvent system, the conversion increased markedly with the elongation of the chain length of the vinyl ester, from 43.2% for vinyl caprate to 99.6% for vinyl stearate (Figure 6A). When sugar esters were produced by Novozym 435-catalyzed esterification of methyl glucoside and fatty acids, the yield of this reaction was also closely related to the chain length of the fatty acid. As can be seen from Figure 6B, the conversion increased again with an increase in the fatty acid chain length from C10 to C18, in both the pure 2M2B and IL/2M2B mixtures. The same phenomenon has already been observed by Zhang et al. [35] when carrying out an investigation on esterification of sorbitol and a series of fatty acids (C10—C18) in *tert*-butanol, catalyzed by *Candida sp.* 99–125 lipase; Adelhorst et al. [11] also observed a faster reaction with the longer fatty acids (C12—C18) compared to the shorter ones (C8—C10) in the solvent-free system, by using a set of lipases from different sources as the catalysts, whereas Yang et al. [36] carried out regioselective acylation of helicid with fatty acid vinyl esters in tetrahydrofuran and observed a bell-shaped relationship between the initial reaction rate and the chain length of the vinyl ester.

Our experiment suggests that the chain length of the acyl donor has a significant impact on the SFAE synthesis in the IL system, regardless of the enzyme (i.e., Novozym 435 or Lipozyme TLIM) or the reaction type (i.e., esterification or transesterification) to be used. This seems to be consistent with the general preference of lipases for lipophilic substrates [37]. Part of the reason may also be attributed to a better compatibility of the long alkyl chain of the fatty acid moiety with the enzyme’s active site or with the hydrophobic cation of the IL used in the bisolvent system.

Figure 6B again reveals that introducing the IL as a co-solvent to the reaction system is favorable, in which higher conversions can be obtained.

## 3. Materials and Methods 

### 3.1. Materials

Novozym 435 (*Candida antarctica* lipase, CALB, immobilized on acrylic resins via hydrophobic adsorption) and Lipozyme TLIM (*Thermomyces lanuginose* lipase, TLL, immobilized on a silicate via ionic adsorption) were purchased from Novozymes Investment Co., Ltd. (Beijing, China). Ionic liquids were obtained from ShangHai Cheng Jie Chemical Co., Ltd. (Shanghai, China). Celite^®^ 545 and vinyl laurate were from Sigma-Aldrich China Inc., (Shanghai, China), while other vinyl esters were from TCI Development Co., Ltd., (Shanghai, China). α-d-Glucose (Glc), α-d-methylglucoside (Me-Glc), and all other reagents used were of analytical grade from local manufacturers.

### 3.2. Preparation of DESs and Determination of Their Water Contents and Viscosities

The 24 DESs were prepared by mixing two cholinium salts (ChCl and ChAc) with four H-bond donors (HBDs) (urea, glycerol, acetamide, and ethylene glycol) respectively at three molar ratios (1:2, 1:1, and 2:1), as described in [28]. For viscosity measurements, the water contents of all DESs were first determined via Karl–Fischer titration with an 831 KF coulometer (Metrohm, Herisau, Switzerland), and extra water was added until reaching a final water content of 10% *w/w*. The viscosity was then measured at 40 °C by using an AR1000 rheometer (TA Instruments, New Castle, DE, USA).

### 3.3. Lipozyme TLIM-Catalyzed Transesterification of α-d-Glucose and Vinyl Esters

A typical reaction was carried out by adding 0.054 g of glucose (corresponding to 0.3 mol/L of the reaction system, only partially dissolved) to a 5 mL capped test tube containing 0.3 M of vinyl ester (totally dissolved) and 100 mg of molecular sieves (4 Å) in 1mL of solvent (pure IL or IL/2M2B mixture). Lipozyme TLIM (20 mg) was added, and the tube was placed in an incubator/shaker with an agitation of 300 rpm at 40 °C to start the reaction. Periodically, a 10 µL sample was taken and 3 times diluted with DMSO for High performance liquid chromatography (HPLC) analysis as indicated below. The conversion was calculated as the percentage molar ratio of the ester produced to the total glucose added to the reaction system. All tests throughout this study were performed at least three times subjected to an error less than 10%, and the results presented are the means of the replicate assays.

### 3.4. Novozym 435-Catalyzed Esterification of Methyl Glucoside and Fatty Acids

Novozym 435 (normally 10 mg) was added to a 5-mL capped test tube containing methyl glucoside (0.058 g, corresponding to 0.3 mol/L of the reaction system, only partially dissolved), a fatty acid (0.3 M), and molecular sieves (150 mg) in 1 mL of solvent (pure 2M2B or IL or IL/2M2B mixture), which was agitated at 300 rpm and 45 °C. Periodically, a 10-µL sample was taken and 3 times diluted with DMSO for HPLC analysis as indicated below. The conversion was calculated as in Section 3.3, based on the total amount of methyl glucoside added to the reaction system.

### 3.5. HPLC Analysis

A Shimadzu LC-20AT HPLC system equipped with a refractive index detector (Shimadzu RID-10A, Kyoto, Japan) and a 150 × 4.6 mm, 5-μm inertsil ODS-SP column (GL Sciences Inc., Torrance, CA, USA) was used for HPLC analysis. A 10 µL sample was injected, and a solvent mixture of methanol and water was employed as the mobile phase with a flow rate of 1.0 mL/min, operated at 40 °C. Water adjusted to pH 3.5 with acetic acid was mixed with methanol to form the mobile phase at 25:75 *v/v* for reactions with vinyl caprate or capric acid as the co-substrate, at 15:85 *v/v* for reactions with vinyl laurate, vinyl myristate or their acids, and at 10:90 *v/v* for reactions with vinyl palmitate, vinyl stearate, or their acids.

### 3.6. RSM Experimental Design

A 4-factor-3-level Box-Behnken design of response surface methodology was carried out using Design-Expert v8.0.6, DOE software developed by Stat-Ease, Inc. (Minneapolis, MN, USA). The four factors to be selected for optimization were reaction time, enzyme dosage, VL/Glc molar ratio, and 2M2B/IL volumetric ratio. The obtained conversion was taken as a response parameter for the model. Experimental results were analyzed by applying the ANOVA (analysis of variance) technique implemented in the Design-Expert software. RSM Data can be found in the Supplementary Materials.

## 4. Conclusions 

In this study, two synthetic reactions (i.e., esterification and transesterification) to produce SFAEs, catalyzed by two lipases (i.e., Novozym 435 and Lipozyme TLIM), were investigated by screening different ILs and DESs as a reaction medium, by comparing methyl glucoside vs. glucose as the starting material, and by studying the effect of the chain length of the fatty acid moiety on the production yield. As compared to the use of pure IL or 2M2B as the reaction medium, the [HMIm][TfO]/2M2B bisolvent system has been demonstrated to be a favorable option for both reactions catalyzed by the two enzymes, leading to the achievement of superior specific productivities (769.6 and 397.5 µmol/h/g, respectively), which are much higher than those reported in the literature.

## Figures and Tables

**Table 1:** Conversions (%) obtained by Lipozyme TLIM-catalyzed transesterification and Novozym 435-catalyzed esterification in 14 different ionic liquids (ILs).

No.	Ionic Liquid ^a^	Transesterification ^b^	Esterification ^c^
1	[BMIm][PF_6_]	0.20	6.21
2	[BMIm][BF_4_]	2.32	22.46
3	[EMIm][MeSO_4_]	0.20	0.21
4	[EMIm][MeSO_4_]	0.20	0.21
5	[BMIm][MeSO_4_]	0.60	0.21
6	[EMIm][Tf_2_N]	2.11	
7	[BMIm][Tf_2_N]	2.08	5.71
8	[C_7_-MIm][Tf_2_N]	0.20	7.29
9	[C_12_-MIm][Tf_2_N]	0.20	6.59
10	[BTMA][Tf_2_N]	0.20	3.74
11	[HTMA][Tf_2_N]	0.59	3.47
12	[EMIm][TfO]		2.38
13	[BMIm][TfO]	26.50	30.89
14	[HMIm][TfO]	26.84	30.67

^a^ Abbreviations for IL cations: EMIm = 1-ethyl-3-methylimidazolium, BMIm = 1-butyl-3-methylimidazolium, C_7_-MIm = 1-heptyl-3-methylimidazolium, C_12_-MIm = 1-dodecyl-3-methylimidazolium, BTMA = butyltrimethylammonium, HTMA = hexyltrimethylammonium; abbreviations for IL anions: PF_6_ = hexafluorophosphate; BF_4_ = tetrafluoroborate; Tf_2_N = bis(trifluoromethylsulfonyl) imide; TfO = trifluoromethylsulfonate; MeSO_4_ = methylsulfate. ^b^ Conditions used for transesterification reactions: A reaction mixture containing 0.054 g of glucose, 0.3 M of vinyl laurate, 100 mg of molecular sieves, and 20 mg of Lipozyme TLIM in 1 mL of solvent was placed in an incubator/shaker with an agitation of 300 rpm at 60 °C for 24 h. ^c^ Conditions used for esterification reactions: A reaction mixture containing 0.058 g of methyl glucoside, 0.3 M of lauric acid, 100 mg of molecular sieves, and 20 mg Novozym 435 in 1 mL of solvent was placed in an incubator/shaker with agitation of 300 rpm at 45 °C for 24 h.

**Table 2:** Solubility (mg/mL) of glucose and methyl glucoside in [HMIm][TfO]/2M2B mixtures at room temperature (~25 °C) ^a^.

Reagent	IL/2M2B (*v/v*)		
	0/10	1/9	3/7
Glucose	2.5	3.5	7.0
Methyl glucoside	10	12	20

^a^ A certain amount of the substrate was incubated via stirring in the solvent for 30 min, and the solubility was estimated by measuring the amount of the substrate that can be completely dissolved.

**Table 3:** Variables and levels used for the Box–Behnken design.

Variable	Symbol	Level		
		−1	0	+1
Enzyme dosage (mg)	*A*	5	20	35
VL/Glc molar ratio	*B*	0.5	1	1.5
Reaction time (h)	*C*	18	22	26
2M2B/IL (*v/v*)	*D*	1.0	2.5	4.0

## Supplementary Materials

Supplementary materials can be accessed at: http://www.mdpi.com/1420-3049/21/10/1294/s1.
