# Supplementary material for: Enzymatic Synthesis of Glucose-Based Fatty Acid Esters in Bisolvent Systems Containing Ionic Liquids or Deep Eutectic Solvents
Source: Molecules. 2016 Sep 27;21(10):1294. doi: 10.3390/molecules21101294 (PMC6273948; doi:10.3390/molecules21101294)
Supplement: Supplementary file 1 [file molecules-21-01294-s001.pdf]

Supplementary Materials: Enzymatic Synthesis of Glucose-Based Fatty Acid Esters in Bisolvent Systems Containing Ionic Liquids or Deep Eutectic Solvents

Kai-Hua Zhao, Yu-Zheng Cai, Xiao-Sheng Lin, Jun Xiong, Peter J. Halling and Zhen Yang

Std	Run	A:enzyme amount (mg)	B: VL/Glc molar ratio	C: time (h)	D:2M2B/IL v/v ratio	Conversion (%)
1	1	5	0.5	22	2.5	46.428501
16	2	20	1.5	26	2.5	70.7021043
18	3	35	1	18	2.5	68.8598515
27	4	20	1	22	2.5	64.3616164
30	5	20	1	22	2.5	62.2728627
13	6	20	0.5	18	2.5	42.0293115
5	7	20	1	18	1	44.3057805
7	8	20	1	18	4	56.2489272
11	9	5	1	22	4	22.7574848
10	10	35	1	22	1	71.3492678
2	11	35	0.5	22	2.5	55.9538965
8	12	20	1	26	4	66.6535857
3	13	5	1.5	22	2.5	21.7219426
17	14	5	1	18	2.5	14.523895
23	15	20	0.5	22	4	50.4500922
6	16	20	1	26	1	49.6690181
24	17	20	1.5	22	4	69.191572
9	18	5	1	22	1	18.7829106
21	19	20	0.5	22	1	41.5719408
26	20	20	1	22	2.5	65.169174
25	21	20	1	22	2.5	64.174174
14	22	20	1.5	18	2.5	55.5410012
12	23	35	1	22	4	87.7568039
19	24	5	1	26	2.5	22.7333814
4	25	35	1.5	22	2.5	97.9392
28	26	20	1	22	2.5	66.9967629
22	27	20	1.5	22	1	60.1277733
20	28	35	1	26	2.5	98.8052172
29	29	20	1	22	2.5	75.5565667
15	30	20	0.5	26	2.5	52.7394192

Figure S1. Response Surface Methodology (RSM) Experimental Design Data.
